# Should we support prophylactic intervention for asymptomatic kidney stones? A retrospective cohort study with long-term follow-up

**DOI:** 10.1007/s00240-022-01331-4

**Published:** 2022-05-27

**Authors:** Tao Wu, Zhiwei Liu, Shanjin Ma, Wei Xue, Xiaoye Jiang, Jianjun Ma

**Affiliations:** Department of Urology, Tangdu Hospital, Air Force Medical University, 1 Xinsi Rd, Xi’an, 710038 China

**Keywords:** Asymptomatic, Kidney stone, Prophylactic intervention, Surveillance, Follow-up

## Abstract

The purpose of this study was to evaluate the long-term outcomes of patients undergoing prophylactic intervention or active surveillance for asymptomatic kidney stones and identify factors influencing the outcomes. In this retrospective cohort study, we reviewed the medical records of patients with asymptomatic kidney stones in two institutes between November 2014 and November 2019. Standardized questions were asked via phone calls to supplement the outcomes. Pain, hydronephrosis, stone growth, serious infection, gross hematuria, and spontaneous passage were defined as stone-related events. Future intervention was also recorded to evaluate management. A total of 101 patients with 120 kidney units were enrolled in this study. The median follow-up time was 63 months. The patients were classified into the control group (79 cases) or exposure group (41 cases) according to whether they underwent prophylactic intervention before any stone-related events. Generally, the rates of stone-related events and future intervention were significantly different between the two groups (57.0 vs. 12.2%, *p* < 0.001; and 31.6 vs. 4.9%, *p* = 0.002, respectively). After applying stabilized inverse probability of treatment weighted, Cox regression suggested that patients who underwent prophylactic intervention were less likely to experience stone-related events and future intervention (HR = 0.175, and HR = 0.028, respectively). In conclusion, patients who underwent prophylactic intervention had a lower risk of stone-related events and future intervention, although they had some slight complications.

**Supplementary Information:**

The online version contains supplementary material available at 10.1007/s00240-022-01331-4.

## Introduction

Urolithiasis is one of the most prevalent and recurrent urologic diseases. The prevalence of urolithiasis has been estimated to be 5.8–12% in the adult population [1–3]. With advances in imaging technology and more frequent physical examinations, the detection of asymptomatic kidney stones has increased. The prevalence of asymptomatic stone disease is estimated to be between 8.5 and 32.6% [1, 4–6].

The two options for dealing with asymptomatic kidney stones are active surveillance and prophylactic intervention. The latest European Association of Urology and American urological association guidelines for urolithiasis recommended active surveillance for asymptomatic calyceal stones with only a low level of confidence [7, 8]. These guidelines acknowledge that for patients with asymptomatic stones in certain situations (patients with certain professions or poor access to medical care), treatment may be more appropriate. Past studies involving follow-up after the treatment of asymptomatic kidney stones have produced conflicting results [9–11]. However, few studies have been published on prophylactic intervention, particular in the modern era during which endoscopy technology and quality of life have improved.

Recently, COVID-19 has resulted in great changes to our medical condition. In the COVID-19 era, patients experienced more additional examinations along with higher rates of conservative approaches and emergency admissions compared to before COVID-19 [12]. Furthermore, patients undergoing urgent surgery have significantly higher rates of morbidity and mortality than those undergoing elective procedures [13]. This suggests that we should reconsider our management of asymptomatic kidney stones and learn more about the outcomes of prophylactic intervention. In this study, we compared the outcomes associated with prophylactic intervention and active surveillance in patients with kidney stones and identified the factors influencing the outcomes.

## Materials and methods

### Data collection

This was a retrospective cohort study conducted in line with the STROCSS criteria [14]. Approval for this study was granted by the local Institutional Ethics Committee. The patients enrolled in this study were hospitalized in the Tangdu Hospital and Air Force Medical Center between November 2014 and November 2019. For patients with stones in both kidneys, the two kidneys were considered as two separate cases.

The inclusion criteria for this study were as follows: patients with kidney stones occasionally detected by non-contrast CT (NCCT) or ultrasound (US); stone size smaller than 15 mm; no associated symptoms, such as pain in the same laterality, recurrent hematuria, and infection; no co-existing ureteric stones or obstructive stones in the same laterality; and a follow-up period of at least 2 years. The exclusion criteria were as follows: uncertain stone-related events; urinary tract congenital abnormalities; residual fragments following previous treatment; and no medical examination for over 2 years. Our research included newly diagnosed patients and those who were already on active surveillance where data were only collected for the set study period. The exposure group included patients who underwent prophylactic intervention during the study period (including ESWL, RIRS, or PCNL before any stone-related events occurred), while patients who were hospitalized in the same period and underwent active surveillance served as the control group.

The patients were advised to undergo active surveillance or prophylactic intervention based on the patient’s willingness, the characteristics of the kidney stone, and their profession. Pilots are prohibited from flying if physicians think their kidney stones may affect flight safety; thus, many pilots undergo prophylactic intervention. Some patients received pharmacological treatments after the kidney stones were found. The majority (85.8%) of the follow-up US or NCCT examinations were conducted in our hospital; others were conducted at their referring qualified hospitals. All US and NCCT examinations were performed by experienced sonographers with intermediate professional titles or above. Stone size (defined as the maximum axial diameter) was based on the size measured by NCCT. The US results were referenced only when NCCT results were lacking. In patients with multiple stones, stone size was defined as the longest axis of the largest stone rather than the cumulative diameter of all stones. In addition to the stone size, the following clinical data were retrospectively collected from medical records: age, gender, body mass index (BMI), associated diseases (e.g., hyperlipidemia), history of urolithiasis, laterality of the stones, multiple stones or simple stone, stone location (lower pole or other location), complications (evaluated by the Clavien–Dindo scale).

### Follow-up protocol

All patients were visited by the same physician over the phone between December 2021 and January 2022. The patients were asked standardized questions regarding the occurrence of stone-related events and were invited to complete the Wisconsin Stone Quality of Life (WISQOL) survey. The primary outcome of the study was stone-related events, including pain (ipsilateral renal colic), hydronephrosis (caused by obstructive stones), stone growth, serious infection (requiring hospitalization), gross hematuria, and spontaneous passage. The secondary outcome of the study was future intervention (when intervention was required after the initial planned treatment or period of active surveillance). Pain was defined as lumbar or abdominal pain that a physician thought was most likely caused by urinary stones. Hydronephrosis and spontaneous passage were confirmed by NCCT or US. To exclude the possible impact of treatment, any stone passage within 6 months after the stone detection in control group or within 12 months after prophylactic intervention was not considered to be spontaneous passage. Serious infection was determined based on hospitalization records. Stone-free was judged by examination within 12 months after the prophylactic intervention, and residual stones of any size were not considered to be stone-free. Stone growth was defined as an increase in stone diameter exceeding 5 mm compared to the first detected diameter. Informed consent was obtained from all participants in the form of an opt-out option over the phone.

### Statistical analysis

Data were entered into Excel^®^ software and analyzed with R statistical programming software v.4.1.3. [R Core Team (2022)]. Two-tailed Student’s *t* test or Mann–Whitney *U* test was used to analyze significant differences between continuous variables according to whether they conformed to the normal distribution. Pearson’s chi-square test was used to compare categorical variables between groups. To ensure reliably comparable cohorts, we used stabilized inverse probability of treatment weighted (IPTW) multivariable Cox proportional hazard regression models to estimate the effect of prophylactic intervention. Factors that satisfied the proportional hazards assumption were evaluated by univariable and multivariable Cox proportional hazard regression models. Hazard ratios (HRs) and 95% confidence intervals (CIs) were estimated for each factor. All two-sided *p* values < 0.05 were considered statistically significant.

## Results

### Patient characteristics

**Fig. 1:**
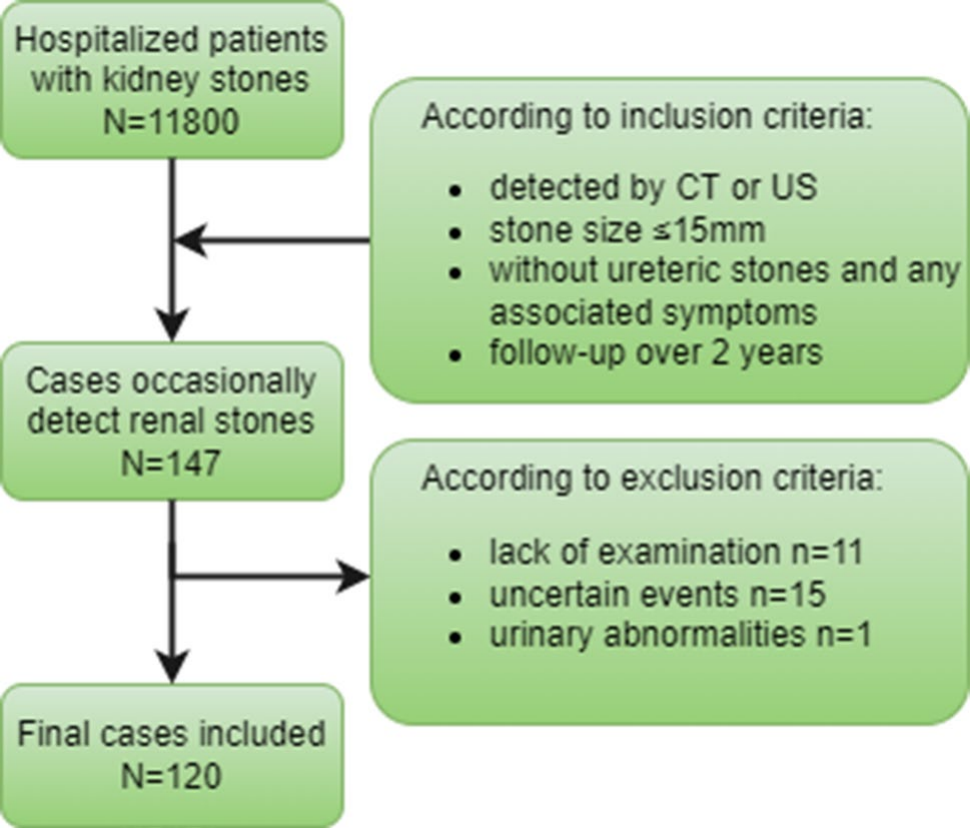
Flow diagram of the study. All 147 kidney units with asymptomatic kidney stones were identified from the medical records of 11,880 patients hospitalized with kidney stones according to the inclusion criteria. Among these patients, 27 kidney units were excluded based on the exclusion criteria. Finally, 120 kidney units in 101 patients were included in the analysis.

From the medical records of 11,880 patients hospitalized with kidney stones, we identified a total of 147 kidney units in 131 patients with asymptomatic kidney stones according to the inclusion criteria. 27 kidney units were excluded based on the exclusion criteria. Finally, 120 kidney units in 101 patients were included in the analysis (Fig. 1). The characteristics of the patients and stones are presented in Table 1. The median age of the patients was approximately 48 years old, and the average BMI was 24.4 kg/m^2^. Most of the patients were men, and over half of the patients were pilots. Patient follow-up was conducted for a median of 63 months with an interquartile range (IQR) of 43.8–78.0 months. Only 35 patients completed the WISQOL survey, and no differences in score were observed between the two groups (*p* = 0.116).

**Table 1:** Characteristics of patients and stones before matching

Variables	All patients (*N* = 120)	Control group (*N* = 79)	Exposure group (*N* = 41)	*p* value
Body mass index (BMI)^a^, kg/m^2^	24.4 ± 2.4	24.7 ± 2.6	23.9 ± 1.9	0.065
Age^b^, years	48.0 (35.3–59.0)	55.0 (42.0–62.0)	37.0 (34.0–46.0)	< 0.001*
Follow-up time^b^	63.0 (43.8–78.0)	68.0 (43.0–82.0)	57.0 (47.0–70.0)	0.106
Stone size^b^, mm	5.0 (3.0–6.0)	4.6 (3.0–6.0)	5.0 (3.5–6.0)	0.305
Sex (male)^c^	102 (85.0%)	66 (83.5%)	36 (87.8%)	0.535
Profession (pilot)^c^	69 (57.5%)	36 (45.6%)	33 (80.5%)	< 0.001*
Multiple stones (yes)^c^	47 (39.2%)	32 (40.5%)	15 (36.6%)	0.676
Location (not lower)^c^	96 (80.0%)	68 (86.1%)	28 (68.3%)	0.021*
Prior history (yes)^c^	27 (22.5%)	21 (26.6%)	6 (14.6%)	0.137
Hyperuricemia (yes)^c^	26 (21.7%)	21 (26.6%)	5 (12.2%)	0.070

*Values are statistically significant

^a^Data presented as mean ± standard deviation, tested by Student’s *t* test

^b^Data presented as median ± interquartile range, tested by Mann–Whitney *U* test

^c^Data presented as frequency (%), tested by Pearson’s chi-square test

The details of the exposure group are shown in Supplementary Table S1. Of the 41 cases in the exposure group, eight underwent two or more prophylactic interventions, and four of them received two kinds of surgery (ESWL and RIRS). Generally, the stone-free rate (SFR) was 58.5% after 12 months. The stone size was reduced by 3.9 mm on average after treatment. The median time from stone detection to prophylactic intervention was 18 days (IQR 9–89 days). Not surprisingly, when the intervention was performed, the increase in stone size compared to the initial state was much lower in the exposure group than in the control group (0 vs. mean 5.9 mm). It should be noted that the number of patients suffered stone-related events was lower in patients with stone-free than in those with residual fragments, and much lower than in control group (4.2 vs. 23.5%, and 4.2 vs. 33.3%, respectively). Otherwise, 36 (87.8%) cases had complications according to the Clavien–Dindo score [15]. Clavien–Dindo grade I and Clavien–Dindo grade II complications were found in 7 (17.1%) and 29 (70.7%) patients, respectively, while no severe complications (Clavien–Dindo grade ≥ III) were found in any patients.

The two groups showed differences in terms of age, profession, and stone location (*p* < 0.001, *p* < 0.001, and *p* = 0.021, respectively). Thus, stabilized IPTW was applied to balance all factors included in the propensity score model (absolute standardized difference < 0.1) except for BMI and hyperuricemia (Supplementary Table S2, and Supplementary Fig. S1). BMI, hyperuricemia, and those factors with *p* values less than 0.1 in univariate analysis were included in the multivariate analysis (Supplementary Table S3).

### Stone-related events

The details of stone-related events are presented in Table 2. Among all patients in this study, 45 (37.5%) had stone-related events. The median time to onset of stone-related events was 15.0 (IQR 9.0–32.5) months. Generally, the incidences of stone-related events, pain, hydronephrosis, and spontaneous stone passage were significantly higher in the control group than in the exposure group (*p* < 0.001, *p* = 0.025, *p* = 0.036, and *p* = 0.002, respectively). Based on the Kaplan–Meier estimator, the rate of stone-related events was significantly different between the two groups (*p* < 0.001).

**Table 2:** Details of Primary and Secondary Outcomes

	Overall (*N* = 120)	Control group (*N* = 79)	Exposure group (*N* = 41)	*p* value
Stone-related events^a^	45 (37.5%)	40 (57.0%)	5 (12.2%)	< 0.001*
Time to events, ^b^months	15.0 (9.0–32.5)	14.5 (9.0–28.8)	36.0 (14.5–43.5)	
Pain	18 (15.0%)	16 (20.3%)	2 (4.9%)	
Hydronephrosis	17 (14.2%)	15 (19.0%)	2 (4.9%)	
Stone growth	10 (8.3%)	8 (10.1%)	2 (4.9%)	
Serious infection	2 (1.7%)	2 (2.6%)	0 (0%)	
Gross hematuria	5 (4.2%)	4 (5.1%)	1 (2.4%)	
Spontaneous passage	16 (13.3%)	16 (20.3%)	0 (0%)	
Future intervention^a^	25 (20.8%)	23 (31.6%)	2 (4.9%)	0.002*
Time to intervention, ^b^months	24.0 (9.0–34.0)	19.0 (9.0–28.0)	37.5 (31.0–44.0)	

*Values are statistically significant

^a^Data presented as frequency (%)

^b^Data presented as median (interquartile range)

**Fig. 2:**
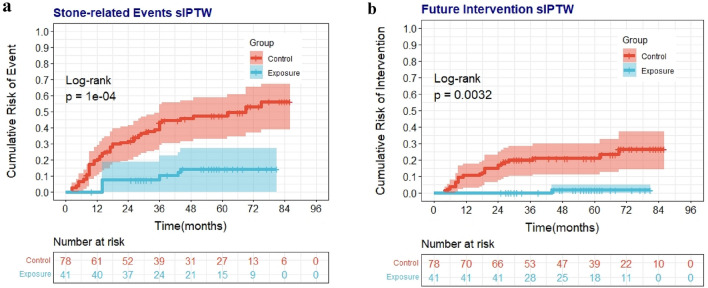
The cumulative risk of outcomes. The cumulative risk of stone-related events and future intervention were significantly higher in the control group than in the exposure group (*p* = 0.0001, and *p* = 0.0032, respectively).

After stabilized IPTW was applied, multivariate analysis showed that the patients in the exposure group had a lower risk of stone-related events compared with patients in the control group (HR = 0.175, 95% CI = 0.057–0.539; Table 3, and Fig. 2a). Stone size greater than 5 mm and hyperuricemia were significant factors influencing the occurrence of stone-related events (HR = 2.545, 95% CI = 1.247–5.196, and HR = 3.388, 95% CI = 1.759–6.527, respectively).

**Table 3:** Multivariate analysis of the primary and secondary outcomes

Variables	Stone-related events		Future intervention	
	HR (95% CI)	*p* value	HR (95% CI)	*p* value
Intervention	0.175 (0.057–0.539)	0.002*	0.028 (0.003–0.255)	0.002*
BMI	1.050 (0.927–1.190)	0.443	1.139 (0.926–1.400)	0.218
Stone size (> 5 mm)	2.545 (1.247–5.196)	0.010*	3.207 (1.286–7.996)	0.012*
Sex (male)	0.523 (0.218–1.253)	0.146	0.454 (0.168–1.227)	0.120
Profession (pilot)	0.614 (0.288–1.307)	0.206	0.224 (0.073–0.685)	0.009*
Location (not lower)	–		2.299 (0.325–16.262)	0.404
Prior history (yes)	1.420 (0.756–2.664)	0.275	–	
Hyperuricemia (yes)	3.388 (1.759–6.527)	< 0.001*	1.361 (0.481–3.853)	0.562

*Values are statistically significant

### Future intervention

Among all patients, 25 (20.8%) received intervention after the initial treatment or surveillance period, and the median time to onset of intervention was 19 (IQR 9–28) months (Table 2). The future intervention rate in the exposure group was much lower than that in the control group (*p* = 0.002), in agreement with the Kaplan–Meier analysis results (*p* = 0.005). Specifically, two patients with residual fragments required future intervention, while no stone-free patients required future intervention.

To accurately analyze the variables associated with stone-related intervention, the kidney units of patients who underwent surgery entirely based on the patient’s choice rather than actual stone-related events were not considered as requiring intervention. Among all kidney units, four units were not considered to require intervention as they did not represent a stone-related event.

The factors affecting the likelihood of stone-related intervention were identified by multivariate Cox regression after applying stabilized IPTW. The prophylactic intervention, stone size, and profession were significant factors for future intervention (HR = 0.028, 95% CI = 0.003–0.255; HR = 3.207, 95% CI = 1.286–7.996; HR = 0.224, 95% CI = 0.073–0.685; Table 3, and Fig. 2b).

## Discussion

Many studies have been published about patients with asymptomatic kidney stones undergoing active surveillance. However, research about patients receiving prophylactic intervention for kidney stones remains insufficient. Living with kidney stones may cause health problems. For example, kidney stones were found to double the risk of papillary renal cell carcinoma and increase the risk of upper tract urothelial carcinoma by 66% [16]. Recurrent symptomatic stones increase the risk for kidney failure by two times [17]. In addition, in many cases, patients are reluctant to follow instructions or prefer prophylactic intervention [18–20]. These factors make active surveillance less effective than expected. Therefore, the outcomes of prophylactic intervention should be further investigated. In this study, we evaluated the long-term outcomes of prophylactic intervention to explore whether asymptomatic kidney stones should be treated before symptom progression.

For patients undergoing active surveillance for asymptomatic kidney stones, the rates of stone-related events and need for intervention have been reported to be 15.3–61.8% [21–24]. Among all kidney units in patients undergoing active surveillance in the present study, stone-related events occurred in 57.0 and 31.6% required intervention. The rates of serious infection and gross hematuria in this study were low, likely due to the strict inclusion criteria and recall bias, which may cause mild symptoms to be overlooked.

Yuruk et al. prospectively evaluated 94 patients who underwent prophylactic intervention for asymptomatic lower-pole renal calculi. The mean follow-up period was 19.3 (range 12–29) months. The results indicated that patients should be informed in detail about all management strategies; in particular, PCNL resulted in a 100% SFR and less renal scarring compared to ESWL. Using a similar method, Sener et al. [11] evaluated 150 patients and reported SFRs exceeding 90% from RIRS and ESWL. The authors concluded that observation is an option for managing asymptomatic lower-pole kidney stones because the patients are likely to be event free for a prolonged period. The SFR in our study was lower than previously reported values, likely due to the strict definition of stone-free; in Sener et al., residual fragments smaller than 3 mm were deemed to be clinically insignificant and were not processed further, whereas these fragments were recorded as residual stones in our study.

In this study, the increase in stone size between the initial state and surgical intervention was much smaller in the exposure group than in the control group, confirming that prophylactic intervention can address stones when the stone size was relatively small. Slight complications were reported after most prophylactic interventions, but no patients required additional surgery for complications, consistent with other reports [25–27].

Few studies have reported follow-up outcomes in patients receiving prophylactic intervention for asymptomatic kidney stones. Li et al. [28] reported that 41.7% of patients who underwent bilateral surgery required future intervention, while the rate in patients who underwent ipsilateral surgery with asymptomatic concurrent contralateral stones was 43%. The authors concluded that compared with prophylactic intervention, observation did not increase the risk of future intervention, except for stone size greater than 6 mm. In contrast, we estimated a considerably lower future intervention rate and drew a different conclusion. Compared with patients under active surveillance, asymptomatic patients who received prophylactic intervention had a significantly lower cumulative risk of future intervention and stone-related events. The primary reason may be that our study was strictly limited to non-symptomatic kidney stones before prophylactic intervention. Moreover, the stone size in our study was smaller. Chew et al. [29] reported that patients with residual fragments smaller than 4 mm were less likely to require future intervention than those larger than 4 mm. Therefore, our data are not comparable with those of Li et al.

Identifying the risk factors for stone-related events and future intervention can be helpful for clinical management. Age, annual stone growth, stone size, stone location, hyperuricemia, and prior stone history are associated with a greater risk of symptom progression [21, 23, 24]. In our study, compared to patients in control group, patients in the exposure group had a 17.5% risk of stone-related events and only a 2.8% risk of future intervention after the initial treatment. Thus, prophylactic intervention may extend the nonevent duration. Stone size greater than 5 mm increased the risk for stone-related events and future intervention by over two times compared to smaller stones, consistent with other studies [24]. Patients with hyperuricemia were more likely to suffer stone-related events. Pilots may have less future intervention.

We acknowledge that our study has several limitations. First, as a retrospective study, data were collected from electronic medical records and supplemented by phone calls. Thus, recall bias may have affected the results. However, severe symptoms are easy to recall and were likely to be reported. The partial neglect of mild symptoms would not cause major problems in the results. Second, this study did not include long-term complications or any assessment of dietary factors. Many patients did not complete the examinations and surveys for economic reasons or just to avoid trouble. Thus, we may have overestimated the advantages of prophylactic intervention. Third, the sample size in this study, particularly for the exposure group, was small, and sample bias may have been a factor. A multicenter, prospective study with a larger simple size is needed to confirm our results.

## Conclusion

Compared with those receiving active surveillance, patients who underwent prophylactic intervention had lower risks of stone-related events and future intervention. Therefore, more patients, especially those with risk factors, poor medical condition, or specific professions, should be recommended for prophylactic intervention to prevent the situation from becoming worse.

## Supplementary Information

Below is the link to the electronic supplementary material.

Supplementary file1 (PDF 254 KB)

## Data Availability

The datasets generated during the current study are available from the corresponding author on reasonable request.
