# Supplementary material for: Should we support prophylactic intervention for asymptomatic kidney stones? A retrospective cohort study with long-term follow-up
Source: Urolithiasis. 2022 May 27;50(4):431–7. doi: 10.1007/s00240-022-01331-4 (PMC9137265; doi:10.1007/s00240-022-01331-4)
Supplement: Supplementary file 1 — Supplementary file1 (PDF 254 KB) [file 240_2022_1331_MOESM1_ESM.pdf]

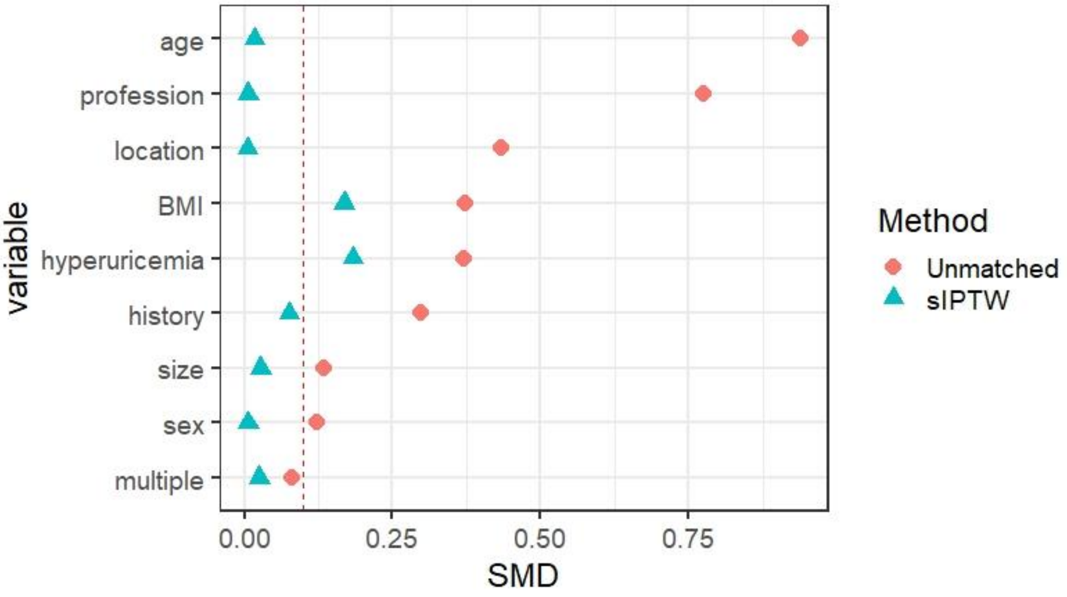

Fig. S1 Balanced diagnostic plot of baseline factors before and after stabilized IPTW

Table S1 Details of Exposure Group

	Stone-free N=24	Residual fragments N=17
**Prophylactic intervention**		
ESWL[a]	4(17%)	2(12%)
RIRS[a]	19(79%)	12(71%)
PCNL[a]	1(4%)	3(18%)
**Twice or more**[a]	2(8%)	6(35%)
**Two kinds of intervention**[a]	1(4%)	3(18%)
**Time to intervention,**[b] **days**	16(9-68)	19(8-130)
**Mean stone size,**[c] **mm**		
Before intervention	5.0±2.6	6.2±3.7
After intervention	0	4.0±2.0
Maximum diameter reduction	5.2±2.6	2.2±3.4
**Complications**[a]		
Overall	20(83%)	16(94%)
Clavien I	3(13%)	4(24%)
Clavien II	17(71%)	12(71%)
≥Clavien III	0	0
**Stone-related events after treatment**[a]	1(4%)	4(24%)
**Time to events after treatment,**[b] **months**	36	29(15-43)
**Future intervention**[a]	0(0%)	2(12%)

a. Data presented as frequency (%)

b. Data presented as median (interquartile range)

c. Data presented as mean ± standard deviation

Table S2 Demographics and Baseline Disease Characteristics, Before and After Stabilized Inverse Probability of Treatment Weighting

Variables		Before			After		
		Control group N=79	Exposure group N=41	*p* value	Control group N=78.2	Exposure group N=40.6	*p* value
**Sex (%)**	**Female**	13 (16.5)	5 (12.2)	0.726	11.9 (15.2)	6.3 (15.5)	0.977
	**Male**	66 (83.5)	36 (87.8)		66.3 (84.8)	34.3 (84.5)	
**Profession (%)**	**Other**	43 (54.4)	8 (19.5)	0.001*	33.9 (43.4)	17.5 (43.1)	0.982
	**Pilot**	36 (45.6)	33 (80.5)		44.2 (56.6)	23.1 (56.9)	
**Multiple stone (%)**	**No**	47 (59.5)	26 (63.4)	0.826	48.3 (61.8)	25.6 (63.0)	0.922
	**Yes**	32 (40.5)	15 (36.6)		29.9 (38.2)	15.0 (37.0)	
**Location (%)**	**Lower**	11 (13.9)	13 (31.7)	0.039*	16.0 (20.5)	8.2 (20.3)	0.979
	**Not**	68 (86.1)	28 (68.3)		62.1 (79.5)	32.4 (79.7)	
**Size (%)**	**<5 mm**	40 (50.6)	18 (43.9)	0.612	40.1 (51.2)	20.2 (49.8)	0.917
	**≥5 mm**	39 (49.4)	23 (56.1)		38.1 (48.8)	20.4 (50.2)	
**Stone history (%)**	**No**	58 (73.4)	35 (85.4)	0.209	60.2 (76.9)	29.9 (73.7)	0.779
	**Yes**	21 (26.6)	6 (14.6)		18.0 (23.1)	10.7 (26.3)	
**Hyperuricemia (%)**	**No**	58 (73.4)	36 (87.8)	0.114	61.5 (78.7)	34.8 (85.7)	0.465
	**Yes**	21 (26.6)	5 (12.2)		16.6 (21.3)	5.8 (14.3)	
**Age (Mean(SD))**		53.10 (14.18)	41.12 (11.16)	<0.001*	49.32 (14.38)	49.58 (14.70)	0.955
**BMI (Mean (SD))**		24.74 (2.59)	23.88 (1.94)	0.065	24.55 (2.49)	24.13 (2.50)	0.592

* Values are statistically significant

Table S3 Univariate analysis of stone-related events and future intervention after sIPTW

variables	Univariate analysis for stone-related events		Univariate analysis for future intervention	
	HR（95%CI）	*p* value	HR（95%CI）	*p* value
**Intervention**	0.203(0.061-0.670)	0.009	0.042(0.005-0.326)	0.002
**BMI**	1.051(0.914-1.210)	0.485	1.064(0.843-1.342)	0.603
**Age**	1.001(0.977-1.026)	0.909	0.996(0.964-1.029)	0.820
**Sex (male)**	0.388(0.197-0.766)	0.006	0.299(0.107-0.839)	0.022
**Profession (pilot)**	0.455(0.221-0.934)	0.032	0.225(0.075-0.669)	0.007
**Multiple stones (yes)**	0.692(0.331-1.444)	0.327	0.505(0.189-1.350)	0.173
**Location (not lower)**	1.629(0.592-4.483)	0.344	4.002(0.856-18.718)	0.078
**Stone size (≥5 mm)**	2.003(0.981-4.090)	0.056	2.956(1.058-8.263)	0.039
**Prior history (yes)**	2.072(0.947-4.531)	0.068	1.228(0.436-3.458)	0.697
**Hyperuricemia (yes)**	2.379(1.080-5.240)	0.031	1.258(0.449-3.527)	0.662
